# Specific Serotonergic Denervation Affects tau Pathology and Cognition without Altering Senile Plaques Deposition in APP/PS1 Mice

**DOI:** 10.1371/journal.pone.0079947

**Published:** 2013-11-21

**Authors:** Juan Jose Ramos-Rodriguez, Sara Molina-Gil, Raquel Rey-Brea, Esther Berrocoso, Monica Garcia-Alloza

**Affiliations:** 1 Division of Physiology, School of Medicine, University of Cadiz, Cadiz, Spain; 2 Department of Neuroscience, School of Medicine, University of Cadiz, Cadiz, Spain; 3 Centro de Investigación Biomédica en Red de Salud Mental, Instituto de Salud Carlos III. Madrid, Spain; Nathan Kline Institute and New York University School of Medicine, United States of America

## Abstract

Senile plaques and neurofibrillary tangles are major neuropathological features of Alzheimer's Disease (AD), however neuronal loss is the alteration that best correlates with cognitive impairment in AD patients. Underlying neurotoxic mechanisms are not completely understood although specific neurotransmission deficiencies have been observed in AD patients and, in animal models, cholinergic and noradrenergic denervation may increase amyloid-beta deposition and tau phosphorylation in denervated areas. On the other hand brainstem neurodegeneration has been suggested as an initial event in AD, and serotonergic dysfunction, as well as reductions in raphe neurones density, have been reported in AD patients. In this study we addressed whether specific serotonergic denervation, by administering 5,7-dihydroxitriptamine (5,7-DHT) in the raphe nuclei, could also worsen central pathology in APPswe/PS1dE9 mice or interfere with learning and memory activities. In our hands specific serotonergic denervation increased tau phosphorylation in denervated cortex, without affecting amyloid-beta (Aβ) pathology. We also observed that APPswe/PS1dE9 mice lesioned with 5,7-DHT were impaired in the Morris water maze test, supporting a synergistic effect of the serotonergic denervation and the presence of APP/PS1 transgenes on learning and memory impairment. Altogether our data suggest that serotonergic denervation may interfere with some pathological aspects observed in AD, including tau phosphorylation or cognitive impairment, without affecting Aβ pathology, supporting a differential role of specific neurotransmitter systems in AD.

## Introduction

Major hallmarks of Alzheimer's disease (AD) include amyloid-beta (Aβ) deposition as senile plaques (SP), neurofibrillary tangles composed by hyperphosphorylated tau and neuronal loss [1], however the underlying mechanism as well as the specific relationship between these pathological features remain unclear. Diverse lines of evidence support the role of different forms of Aβ as a central key in neuronal dysfunction observed in AD patients [2], [3]. Also neurofibrillary tangles and dendritic neuropil threads of abnormally phosphorylated tau protein can be observed in neurons inducing alterations [4]. In this sense, 1) misfolding of tau proteins, 2) circuit-based transfer to new cell populations and 3) differentiation induced degeneration could be the sequence of tau-induced neurodegeneration [5]. On the other hand it remains possible that specific neurodegeneration may induce or worsen the pathological features associated with AD, by potentiating Aβ deposition, tau phosphorylation or cognitive impairment [6], [7]. Among others, serotonergic alterations and neurodegeneration have been implicated in cognitive alterations, and low extracellular 5-HT levels seem to be associated with impaired memory consolidation [8]. Following this idea, the role of different serotonergic receptors has been explored since they regulate a wide variety of neurotransmitters such as glutamate or acetylcholine, facilitating or interfering learning and memory processes. It has even been suggested that neurodegeneration in AD could originate in the brainsterm [9]. Following this idea previous studies have shown an increased relative risk of dementia in depressed patients [10], and it seems that behavioural and psychological symptoms, closely related to altered brainstem and serotonergic function, might be detected more than 2 years before AD is diagnosed [11]. In this sense it has also been pointed out that serotonergic activation, through specific serotonergic receptors, may interfere with downstream protein phosphorylation, and this possibility has been suggested as a feasible mechanism for AD pathology [12]. Moreover *postmortem* studies have also detected early phospho-tau neurofibrillary changes in the dorsal raphe nuclei (RN) [13].

APPswe/PS1dE9 mice have been widely used by the scientific community as animal models of AD, since they show SP deposition at 4–6 months of age [14], [15] as well as learning and memory deficits by 8 months [16]. However, as it occurs in similar AD transgenic models, they do not reproduce the complexity of the illness, since they do not show overt neuronal loss or tau pathology [17]. Therefore, producing an animal model that shows both specific brainstem neuronal loss and Aβ deposition could help elucidate the role of the serotonergic system in AD associated pathology. We have selectively removed RN innervations to the hippocampus and cortex by local administration of the selective neurotoxin 5,7-dihydroxytryptamine (5,7-DHT), that can specifically remove ascending serotonergic innervations [18], [19], [20] to cortex and hippocampus, relevant areas in cognition, and severely affected in AD. We have assessed the effect of the lesion on cognition, SP and Aβ levels, as well as on tau phosphorylation, as indicators of the pathology associated with AD. Whereas we did not detect accelerated Aβ pathology in denervated areas under study, we detected an increase in tau phosphorylation, specially evident in the cortex of denervated APPswe/PS1dE9 mice. Moreover we also observed that transgenic lesioned mice were significantly impaired in the acquisition of the Morris water maze, suggesting a specific role of the serotonergic system in the evolution of AD pathology.

## Materials and Methods

### 1. Animals and serotonergic denervation

APPswe/PS1dE9 mice were obtained from Jackson Laboratory. Animals were aged to ∼7 months of age and experimental procedures were approved by the Animal Care and Use Committee of the University of Cadiz, in accordance with the Guidelines for Care and Use of experimental animals (European Commission Directive 86/609/CEE and Spanish Royal Decree 1201/2005).

In order to remove serotonergic innervation to the cortex and hippocampus of APPswe/PS1dE9 mice (n = 10–12/group), the RN were exposed to the specific serotonergic toxin 5,7-dihydroxytryptamine (5,7-DHT) (Sigma, OR, USA). We injected 1 µl of 5,7-DHT (1.6 µg/µl, in 0.2% ascorbic acid, Sigma, OR, USA) as previously described [21], [22], [23]. RN (including the caudal linar nucleus of the raphe, the dorsal raphe nucleus and the median raphe nucleus) were reached by stereotaxic coordinates as follows: AP 4 mm; ML 0 mm from Bregma and DV 4.3 mm from the dura [24]. In order to protect noradrenergic and dopaminergic neurons, mice received desipramin (Sigma) 25 mg/Kg ip and nomifensin (Sigma) 25 mg/kg ip. Sham animals followed the same procedure but received 1 µl injection of 0.2% ascorbic acid.

Lesions follow up included animal weight assessment every other day until sacrifice, 2 weeks after the lesions, when behavioural assessment was completed. Half of animals were perfused with 4% paraformaldehyde and the brains were fixed in paraformaldehyde for immuno- and histochemistry studies. The rest of the animals were overdosed with pentobarbital and brains were fresh frozen for biochemical determinations, prior brain weight determination immediately after sacrifice.

### 2. Open field test

In order to assess possible anxiety-related alterations, as a consequence of the serotonergic lesion, spontaneous locomotor activity and preference for open spaces was tested in the open field test (OFT) as previously described with minor modifications [25]. Briefly mice were recorded for 30 minutes in a 22×44 cm boxes by a camera attached to a computer and the Smart (Panlab, Spain) software. An inner area was defined at 10 cm from the borders, in order to indentify anxiely-like behaviour. Total distance traveled, velocity and percentage of time in the inner area were measured every 5 minutes.

### 3. Morris water maze (MWM)

Learning and memory abilities were analyzed in the MWM test as previously described [26], with minor modification. Experiments commenced 10 days after the lesions, to guarantee that lesions were completely established. The maze consisted of a round tank of water (0.95 m in diameter) with four equal virtual quadrants indicated with geometric cues mounted on the walls. An escape platform was 2–3 cm below the water surface and was camouflaged with calcium carbonate to cloud the water. Water temperature was 21±1°C. A camera was mounted above the maze and attached to a computer and Smart software (Panlab, Spain). Testing was conducted in two phases: acquisition and retention. Acquisition consisted of 4 trials/day for 4 days with the platform submerged. During this phase the platform was located in the quadrant 2. The time limit was 60 s/trial with an intertrial interval of 10 min. If the animal did not find the platform, it was placed on it for 10 s. The retention phase began a day after acquisition testing. It consisted of a single trial with the platform removed. Average times to locate the platform during the acquisition phase, as well as the percentage of total time spent in each quadrant during the retention phase, were determined.

### 4. AChE activity

In order to check the selectivity and possible secondary effects of the lesion with 5,7-DHT on the cholinergic system, AChE was assessed in cortex and hippocampus as previously described with minor modifications [6]. Briefly brain tissue from groups under study was homogenised in 30 volumes of 75 mM saline phosphate buffer (pH 7.4) and 110 µl of acetylthiocholine iodide (Sigma, USA) 0.3 mM, 28 µl of saline phosphate buffer 100 mM (pH 7.4) and 7 µl of tissue homogenate were incubated in a 96 well plate for 8 min at 37°C. By adding 28 µl of sodium dodecyl sulphate (Sigma, USA) 0.2% (w/v) and 28 µl of 5.5′-dithio-(2-bisnitrobenzoico) (Sigma, USA) 0.5% (w/v) the reaction was terminated. Color was measured spectrophotometrically at 420 nm (MQX200R2, Biotek instruments, Burlington VT, USA). All samples were assayed in duplicates. Results were expressed as percentage of those obtained for Sham wildtype animals.

### 5. Tryptophan hydroxylase, Aβ, and cholinacetyltrasnfersare immunohistochemistry

We performed tryptophan hydroxylase immunostaining of the RN as well as cortex and hippocampus to assess serotonergic lesions performed. Briefly 4 sections/animal (30 µm) were blocked in 2.5% BSA for 4 hours and after washing out they were incubated for 48 h at 4°C in 1∶500 anti-tryptophan hydroxylase antibody (Millipore, MA, USA) in normal goat serum (NGS) (1%) and glicine (1%). Sections were incubated in 1∶1000 anti-sheep Alexa Fluor 594 (Molecular Probes, OR, USA). After washing out, tissue was mounted with GVA mounting solution (Invitrogen, OR, USA). Sections were photographed with a Laser Olympus U-RFL-T fluorescent microscope (Olympus, Japan). Images were acquired using MMIcellTools software. The number of tryptophan hydroxylase-positive cells was counted in RN and the number of projections was counted in the cortex and hippocampus. Images were analyzed using Adobe Photoshop and Image J softwares, and data were expressed as percentage of Sham wildtype values.

APPswe/PS1 mice lesioned with 5,7-DHT and sham treated were assessed *postmortem* for Aβ burden in cortex and hippocampus. Immunohistochemistry for Aβ was performed as previously described [6], [27] with minor modifications. PFA-fixed 30 µm sections were washed in PBS and pre-treated with 70% formic acid for 10 min at room temperature. Sections were blocked in 5% normal goat serum (NGS) with 0.5% Triton-X100 for 1 h and incubated with anti- βA17-24 antibody 1∶1000 (4G8, Covance, Spain) in 1% NGS overnight at 4°C. After washing in PBS, sections were incubated with anti-mouse Alexa Fluor 594 1∶1000 (Invitrogen, USA) for 1 h. Tissue was also incubated in 0.005% thioflavin S (Sigma, OR, USA) for 10 min and washed in PBS before mounting in aqueous solution and coverslipping. Sections were photographed with a Laser Olympus U-RFL-T fluorescent microscope (Olympus, Japan). Images were acquired using MMIcellTools software. The number of plaques, plaque size and plaque burden (expressed as percentage of analyzed area) were calculated in cortex and hippocampus using Adobe Photoshop and Image J software for each group under study.

AChE is not an exclusive cholinergic marker since it can be detected also in the synaptic hendidure and cholineceptive neurons. Therefore in order to further detect any possible alterations of the cholinergic system as a consequence of the 5,7-DHT lesions, cortex and hippocampus from all groups under study were immunostained for cholinacetyltransferase (ChAT) as previously described [6]. Sections were washed in PBS and incubated in 70% formic acid at 37°C for 30 min. After washing out, sections were blocked in 5% bovine serum albumin (BSA) and 0.1% triton-X for 1 h at room temperature. Tissue was incubated with primary antibody anti-ChAT (1∶1000) (Millipore, MA, USA) in 2.5% BSA for 72 h at 4°C. Sections were incubated in Alexa fluor 594 anti-rabbit 1∶1000 (Invitrogen, USA) at room temperature for 1 h. Sections were mounted, coverslipped and photographed with a Laser Olympus U-RFL-T fluorescent microscope (Olympus, Japan).

### 6. Aβ ELISA measurements

Soluble and insoluble Aβ40 and Aβ42 were quantified in the cortex and hippocampus from groups under study using colorimetric ELISA kits (Wako, Japan, AB40 ref: 294-62501 and AB42 ref: 290-62601) as previously described, with minor modifications [14]. At each step, homogenization of 5–10 mg of tissue in 50 µl of lysis buffer with inhibitor cocktail (ref: 87787 y 87786 respectively, Thermo Scientific Pierce, Spain) was followed by centrifugation at 14.500 RPM for 12 min at 4°C. Supernatants were retained for soluble Aβ40 and 42 levels. The resultant pellet was then extracted with 50 µl of 70% formic acid (FA) in distilled water and then centrifuged 10 min at 14.500 RPM at 4°C, and this fraction was neutralized with 1M Tris (pH 11). Soluble and insoluble fractions were diluted 1∶300 and 1∶10 respectively. Standard curves were made using human Aβ40 and Aβ42 standards provided in the ELISA kit. Absorbance was measured spectrophotometrically at 450 nm (MQX200R2, Biotek instruments, Burlington VT, USA) and data was expressed as pmol/g wet tissue.

### 7. Tau and phospho-tau levels

Cortical and hippocampal samples from 5,7-DHT treated and Sham mice were homogenized in lysis buffer (Cell Signaling, USA) and supplemented with a protease and phosphatase inhibitor cocktail (Sigma, USA). After sonication the homogenates were centrifuged at 4°C for 5 min at 15000 g. Supernatants were collected and protein concentration determined using the Bradford protein assay (Biorad, Germany). Proteins were separated on 10% acrylamide-bisacrylamide gels, followed by electrophoretic transfer to PVDF membranes (Schleicher & Schuell, NH, USA). Membranes were then immersed in blocking buffer (Invitrogen, OR, USA) for 1 h and incubated 30 min at room temperature with primary antibodies for total tau (1∶1000) (DAKO, Glostrup, Denmark) and overnight at 4°C for anti-phospho-tau antibody (1∶1000) (clon AT8, Fisher Scientific, Waltham, Ma) overnight. Membranes were washed and then incubated with chemiluminescent immunodetection system for mouse and rabbit primary antibodies respectively (Invitrogen, Carlsbad, USA) for 1 hour. Signal was detected using Novex AP Chemiluminescent Substrate (Invitrogen, USA) and Kodak Biomax Light Film (Sigma, USA). Immunoblots were semi-quantified by measuring the optical density (OD) of each protein band on scanned film using the Image J software. Each band was normalized to β-actin (1∶2.500.000) (Sigma, OR, USA) optical density, and phospho-tau/total tau ratio was represented as percentage of sham wildtype mice values.

### 8. Statistical analysis

SPSS 13.0 software was used for all statistical analysis. Student t test for independent samples was used to compare lesioned and sham treated APP/PS1 mice in Aβ immunohistochemistry and hystochemistry studies. One-way ANOVA for independent samples was used for AChE biochemistry, tryptophan hydroxylase immunohystochemistry, western-blot studies for tau, and Aβ ELISA, where four groups were under study (Sham APPswe/PS1dE9, 5-DHT APPswe/PS1dE9, Sham wildtype and 5-DHT wildtype). Two-way ANOVA for repeated measures was used to analyze time evolution of the animals after the lesions, motor activity and anxiety studies, as well as in the Morris water maze experiments.

## Results

### 1. RN infusion with 5,-7-DHT reduces tryptophan hydroxylase-possitive neurons

**Figure 1:**
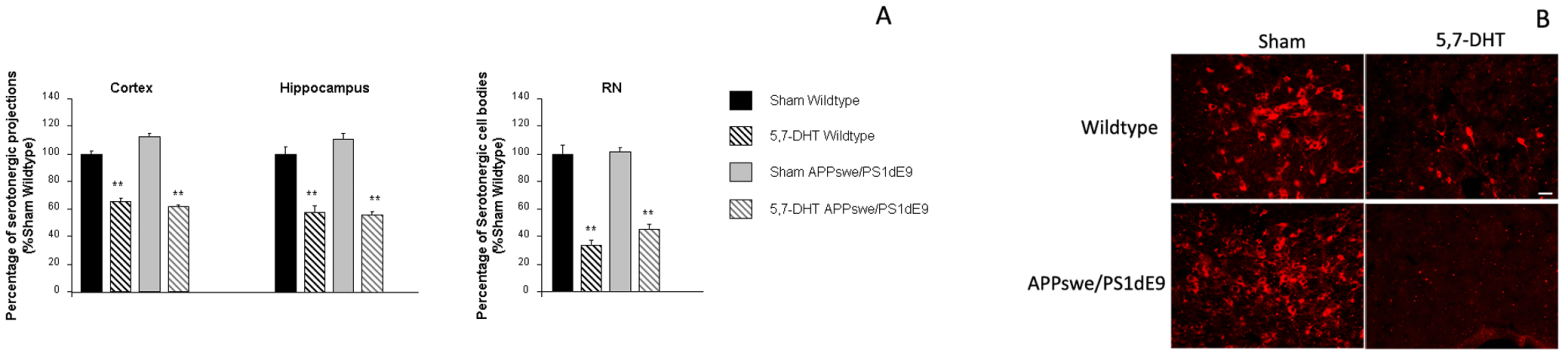
Serotonergic lesions with 5,7-DHT reduce tryptophan hydroxylase immunostaining in RN and denervated areas. **A**) A significant reduction in the number of tryptophan hydroxylase-positive cells is observed in the RN from lesioned mice, when compared with Sham mice. Similarly the number of tryptophan hydroxilase-positive projections was significantly reduced in denervated areas (cortex and hippocampus). Data are expressed as percentage of Sham wildtype mice. Differences were detected by one-way ANOVA followed by Tuckey b test or Tamhane tests as required Cortex [F_(3,304)_ = 13.512, **p<0.01 vs. Sham wildtype and Sham APPswe/PS1dE9], hippocampus [F_(3,99)_ = 52.234 **p<0.01 vs. Sham wildtype and Sham APPswe/PS1dE9], RN [F_(3,99)_ = 52.234 **p<0.01 vs. Sham wildtype and Sham APPswe/PS1dE9]. **B**) Illustrative example of tryptophan hydroxylase immunostaining in the RN from all groups under study, where a significant reduction of tryptophan hydroxylase-positive neurons was observed in 5,7-DHT lesioned mice when compared to Sham treated mice.

Wild-type and APPswe/PS1dE9 mice received injections of the toxin 5,7-DHT into the RN and vehicle-injected animals were used as controls (Sham). Specific assessment of the lesions using tryptophan hydroxylase immunohistochemistry for serotonergic neurons showed a significant reduction of the staining in the RN from lesioned mice 2 weeks after surgery, both APPswe/PS1dE9 and wildtype mice, when compared with Sham treated mice. In the RN from lesioned mice, both wildtype and APPswe/Ps1dE9, reduction of tryptophan hydroxilase-possitive cells reached ∼70% of Sham wildttype values (Figures 1A and 1B). Also, a widespread reduction of tryptophan hydroxylase signal was observed in denervated areas, cortex and hippocampus, when compared with Sham treated mice, reaching ∼50% of Sham treated mice (Figure 1A).

### 2. 5,7-DHT lesions do not affect recovery after surgery or the cholinergic system

**Figure 2:**
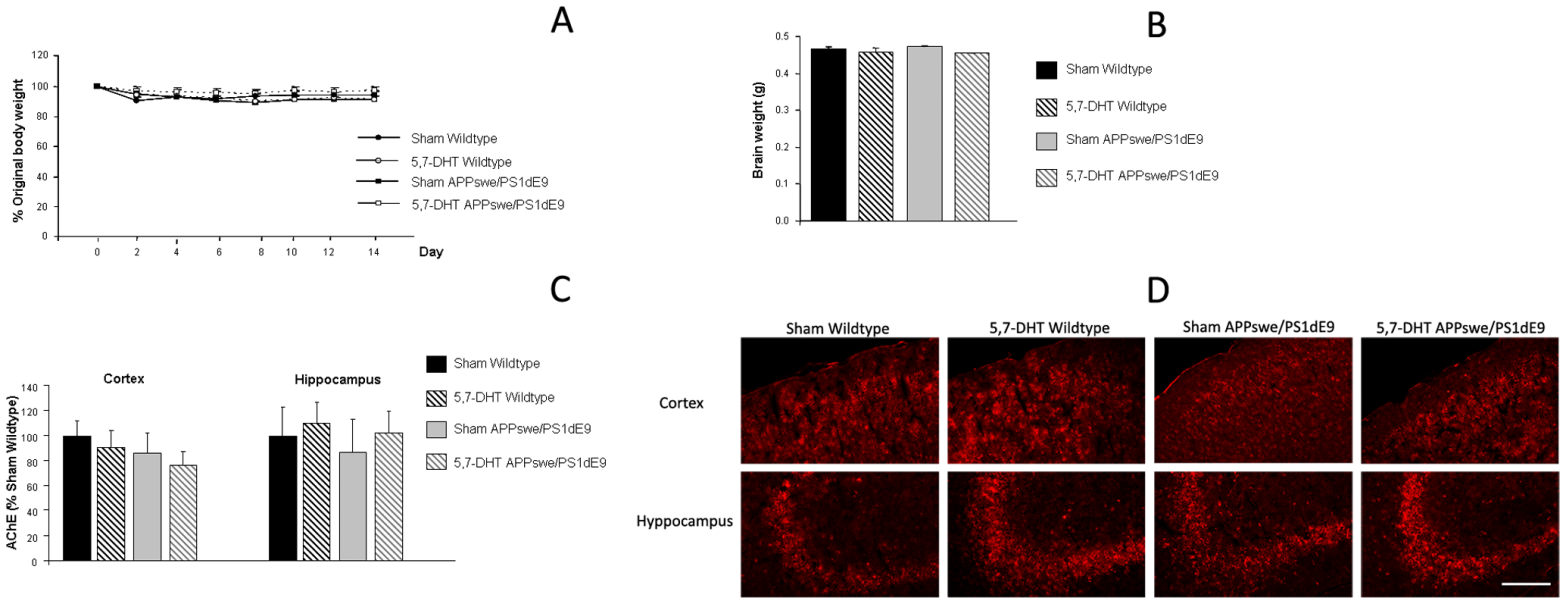
Serotonergic lesions with 5,7-DHT do not affect the recovery after the lesions or the cholinergic system. **A**) Body weight is reduced in all animals after the surgery (expressed as percentage of weight before surgery) although a similar evolution is observed in all groups and no differences were detected by 2-way ANOVA (weightXday) [F_(21,91)_ = 0.980, p = 0.495]. **B**) Similarly brain weight was not affected by 5,7-DHT lesions [F_(3,19)_ = 1.727, p = 0.195]. **C**) AChE activity was not altered in the cortex or hippocampus from wildtype and APPswe/PSdE9 mice lesioned with 5,7-DHT. Data are expressed as percentage of Sham wildtype mice and are representative of 4–5 mice. No differences were detected by one-way ANOVA (Cortex [F_(3, 36)_ = 0.557, p = 0.647] hippocampus [F_(3,32)_ = 0.562, p = 0,644]. **D**) Illustrative example of ChAT immunostaining in ithe cortex and hippocampus (stratum lucidem, CA2 and CA3) where cholinergic cell bodies seem to be preserved after the lesion with 5,7-DHT. Scale bar = 200 µm.

Body weight assessment along the following two weeks, showed a decrease after surgery in all groups under study, and later recovery, whereas no significant weightXday effect was observed by 2 way-ANOVA, when groups under study were compared, supporting that serotonergic lesions did not impair recovery after surgery (Figure 2A). Brain weight was not affected after the lesions, supporting the lack of effect of the lesions on brain atrophy as previously described in selective lesions [6] (Figure 2B). Since there is a close relationship between serotonergic and cholinergic systems we can not exclude that 5,7-DHT serotonergic lesions could interfere with cholinergic neurons in denervated regions. We observed a slight reduction in AChE activity in the cortex from Sham APPswe/PS1dE mice, suggesting some cholinergic damage in this animal model, as previously described [6], [17]. The effect was also observed in 5,7-DHT APPswe/PS1dE mice, however differences did not reach statistical significance in any of the cases (Figure 2C). AChE activity was also preserved in hippocampus from 5,7-DHT treated mice (Figure 2C) suggesting that the cholinergic system is not significantly affected after selective serotonergic denervation of the RN. Since AChE is not exclusively located in cholinergic neurons, ChAT immunostaining was also performed in mice under study and no obvious effect was observed in the cortex or hippocampus from lesioned mice, suggesting that cholinergic system is spared after 5,7-DHT lesions of the RN (Figure 2D).

### 3. Effect of serotonergic denervation on locomotor activity and anxiety-related behavior

**Figure 3:**
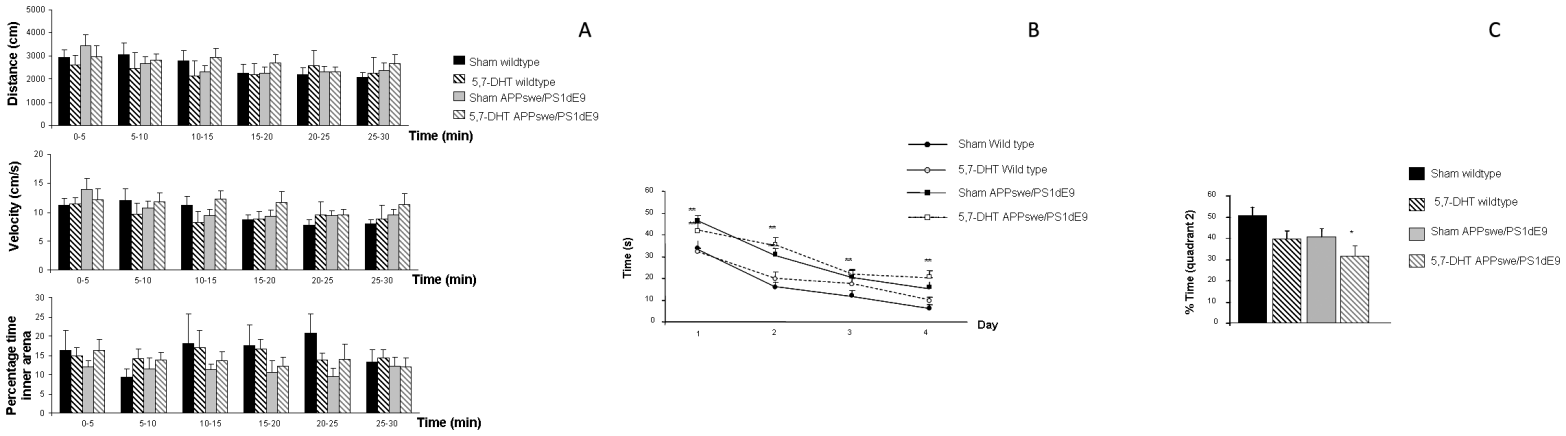
5,7-DHT lesions do not affect motor activity and anxiety behavior whereas learning and memory processes are impaired. **A**) No differences were observed among groups by two-way ANOVA (timexgroup) when total distance travelled [F_(15,65)_ = 1.578, p = 0.105] or velocity were measured [F_(15,65)_ = 1.208, p = 0.289]. No differences were observed in the percentage of time that animals spent in the inner area of the open field [F_(15,63)_ = 0.714, p = 0.761]. **B**) During the acquisition phase in the Morris water maze test we detected a significant treatmentXday effect, and further assessment along individual days, by one-way ANOVA revealed a significant impairment in APPswe/PS1dE9 mice to locate the platform. Day 1: [F_(3,172)_ = 9.213, **p<0.001 vs. sham wildtype and 5,7-DHT wildtype], day 2: [F_(3,172)_ = 16.155, **p<0.001 vs. sham wildtype and 5,7-DHT wildtype], day 3: [F_(3,172)_ = 4.186, **p = 0.007 vs. sham wildtype], day 4: [F_(3,172)_ = 9.329, **p<0.001 vs. sham wildtype and 5,7-DHT wildtype]. **C**) During the retention phase we detected that wildtype lesioned mice and APPswe/PS1dE9 mice spent shorter times in the quadrant wher the platform used to be located (quadrant 2) however one-way ANOVA for independent samples only reached statistical significance in case of 5,7-DHT APPswe/PS1dE9 treated mice [F_(3,37)_ = 2.947, *p = 0.045 vs. sham wildtype].

We did not observe significant differences in the total distance travelled by any of the groups under study at any of the intervals (Figure 3A). Velocity was also similar in all groups suggesting that locomotor activity is not affected after 5,7-DHT lesions (Figure 3A). When we analyzed the percentage of time that animals spent in the inner area, that is separated from the walls by 10 cm, as an indication of the anxiety levels, we did not detect any significant differences, suggesting that local serotonergic lesions of the RN did not significantly affect anxiety-like behaviors (Figure 3A).

### 4. Effect of serotonergic denervation on cognition

Spatial learning and memory abilities were analyzed in all groups using the Morris Water Maze test. We analyzed the time to locate the hidden platform during the acquisition phase and we used two-way ANOVA (groupXday) to detect differences among groups [F_(9,648)_ = 1.914, p = 0.047]. Further daily comparisons were performed with one-way ANOVA followed by Tuckey-b test or Tamhane test as required, showing that transgenic APPswe/PS1dE9 mice were significantly impaired to locate the hidden platform, along the acquisition phase, and that this impairment was slightly worsened after serotonergic denervation (Figure 3B). Similarly, 5,7-DHT treated wildtype mice showed some impairment to locate the platform during the acquisition phase performed slightly worse in the Morris water maze test than wildtype sham mice, however these differences did not reach statistical significance when compared to wildtype sham animals (Figure 3B). In the retention phase, which tests memory preservation, we measured the percentage of time spent by the animals in the quadrant number 2, where the platform was located during the acquisition phase. We observed that wildtype lesioned mice and sham APP/PS1 mice spent shorter times in quadrant number 2, when compared with sham wildtype animals, however these differences only reached statistical significance in case of APPswe/PS1dE9 lesioned mice, suggesting that 5,7-DHT lesions are not aggressive enough to significantly impair learning and memory abilities unless the APP/PS1 transgenes are present (Figure 3C).

When we compared the swimming speed we did not detect any significant differences among the groups under study (sham wildtype: 5.94±0.31 cm/s, 5,7-DHT wildtype: 5.21±0.34 cm/s, sham APPswe/PS1dE9: 5.35±0.23 cm/s, 5,7-DHT APPswe/PS1dE9: 4.91±0.41 cm/s, [F_(3,40)_ = 1.630, p = 0.198]) supporting that observed learning impairment in APPswe/PS1dE9 mice treated with 5,7-DHT, was not due to motor dysfunction in lesioned transgenic mice, as previously observed in the OPT.

### 5. *Postmortem* assessment of Aβ pathology

**Figure 4:**
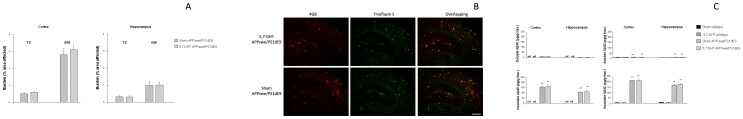
Aβ plaques and levels are not affected in denervated areas after 5,7-DHT administration. **A**) Although a relative increase of thioflavin S was observed in the cortex from APPswe/PS1dE9 lesioned mice, differences did not reach statistical significance when compared with sham treated mice, using Student t test for independent samples (thioflavin S, p = 0.382), and the same pattern was observed after anti-Aβ immunohystochemistry (4G8, p = 0.525). Similarly, no differences were observed in the hippocampus of denervated mice (Thioflavin S p = 0.665; anti-Aβ p = 0.729). **B**) Illustrative example of thioflavin S (green) and anti-Aβ (red) staining in the cortex and hippocampus of Sham APPswe/PS1dE9 mice and 5,7-DHT APPswe/PS1dE9 mice. Scale bar: 250 µm. **C**) Soluble and insoluble Aβ40 and Aβ42 levels were analyzed in the cortex and hippocampus. Since Aβ40 levels were undetectable in wildtype animals, Student t test for independent sample was used to compare Sham and 5,7-DHT APPswe/PS1dE9 mice. One way ANOVA followed by Tuckey b or Tamhane tests was used for the rest of the comparisons, as required. Cortex: Soluble Aβ40 p = 0.924, soluble Aβ42 [F_(3,12)_ = 10.563, **p = 0.001 vs. sham wildtype and 5,7-DHT wildtype], insoluble Aβ40 p = 0.898, insoluble Aβ42 [F_(3,12)_ = 365.243, **p = 0.001 vs. vs. sham wildtype and 5,7-DHT wildtype]. Hippocampus: Soluble Aβ40 p = 0.944, soluble Aβ42 [F_(3,12)_ = 9.215, **p = 0.002 vs. sham wildtype and 5,7-DHT wildtype], insoluble Aβ40 p = 0.804, insoluble Aβ42 [F_(3,12)_ = 165.855, **p<0.001 vs. sham wildtype and 5,7-DHT wildtype].

We assessed the effect of serotonergic denervation of APPswe/PS1dE9 mice on hippocampal and cortical SP deposition by Aβ immunohistochemistry and TS histochemistry. We selected some random sections from wildtype and 5,7-DHT-wildtype treated mice although we did not detect any SP deposited 2 weeks after the lesions. As expected plaques measured with specific 16–27 antibody (4G8) were significantly bigger than labeling after TS staining. Although we observed an slight increase in amyloid burden in the cortex from 5,7-DHT lesioned APPswe/PS1 mice, these differences did not reach statistical significance when compared with sham treated mice, both using TS staining or anti-Aβ immunostaining (Figure 4A and 4B). No differences in Aβ burden were observed when hippocampus was analyzed with TS or 4G8 antibody (Figure 4A and 4B). Plaques sizes were similar after TS staining or 4G8 immunostaining when 5,7-DHT or Sham treated mice were compared (TS Cortex: Sham APPswe/PS1dE9 = 164.08±3.31 µm^2^, 5,7-DHT APPswe/PS1dE9 = 185.11±5.20 µm^2^; 4G8 Cortex: Sham APPswe/PS1dE9 = 275.61±4.95 µm^2^, 5,7-DHT APPswe/PS1dE9 = 317.81±9.28 µm^2^; TS hippocampus: Sham APPswe/PS1dE9 = 171.64±8.30 µm^2^, 5,7-DHT APPswe/PS1dE9 = 157.55±7.29 µm^2^; 4G8 Hippocampus: Sham APPswe/PS1dE9 = 316.37±13.07 µm^2^, 5,7-DHT APPswe/PS1dE9 = 335.21±18.34 µm^2^).

ELISA studies on soluble and insoluble Aβ40 and Aβ42 levels in cortex and hippocampus showed no differences between Sham APPSwe/PS1dE9 mice and 5,7-DHT mice (Figure 4C), supporting the fact that selective serotonergic denervation does not affect Aβ levels.

### 6. Effect of serotonergic lesions on tau phosphorylation

**Figure 5:**
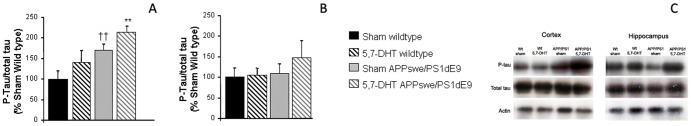
Tau phosphorylation was increased in APPswe/PS1dE9 mice treated with 5,7-DHT. Phosphorylated tau/total tau ratios were expressed as percentage of wild-type Sham values. Differences were detected by one-way ANOVA followed by Tamhane test. **A**) A significant increase in tau phoshporylation was observed in the cortex after serotonergic denervation [F_(3,20)_ = 6.557, **p = 0.03 vs. sham wildtype and 5,7-DHT wildtype, ††p = 0.03 vs. sham wildtype]. **B**) We observed an increase in tau phosphorylation in the hippocampus of APPswe/PS1dE9 mice treated with 5,7-DHT however differences did not reach statistical significance [F_(3, 19)_ = 1.938, p = 0.158]. **C**) Illustrative example of western blot for phospho-tau, total tau and actin, including cortex and hippocampus from sham wildtype, 5,7-DHT wildtype, sham APPswe/PS1dE9 and 5,7-DHT APPswe/PS1dE9 mice. An increase in phospho-tau can be observed in lesioned transgenic mice.

We further analyzed the effect of serotonergic denervation with 5,7-DHT in tau phosphorylation in the cortex and hippocampus of wildtype and transgenic mice. We observed that the cortex was preferentially affected and we detected an increase in tau phosphorylation in wildtype denervated mice, as a consequence of administering the toxin. We also detected an increase in tau phosphorylation when sham APPswe/PS1dE9 mice were anaylized, however, these values were lower than those observed in APPswe/PS1dE9 mice lesioned with 5,7-DHT, suggesting that although serotonergic denervation is enough to alter tau phosphorylation, the effect is worsened by the presence of the APP/PS1 transgenes. [F_(3,20)_ = 6.557, **p = 0.003 vs. sham wildtype and sham APPswe/PS1dE9, ††p = 0.003 vs. sham wildtype] (Figure 5A and 5C). Although in the hippocampus we observed a similar trend, and APPswe/PS1dE9 lesioned mice presented higher levels of phosphorylated tau, differences did not reach statistical significance [F_(3,19)_ = 1.938, p = 0.158] and it remains possible that the serotonergic denervation provoked was more robust in the cortex (Figure 5B and 5C).

## Discussion

Animal models of AD can hardly reproduce the whole range of pathological features, including amyloid pathology, increased tau phosphorylation and synaptic loss observed in humans. Following this idea, APPswe/PS1dE9 mice show early Aβ deposition, by the age of 4 months; though as in other AD transgenic mice, no neuronal loss is spontaneously observed in these animals [17]. In order to study the relationship between these alterations, most of the studies have focused on the effect of Aβ and tau pathology on neuronal morphology and function [2], [3], [5], [28], however approaches trying to establish the role of specific neurotransmitter systems, such as noradrenergic [7] or cholinergic systems [6], on Aβ deposition or tau hyperphosphorylation are more sparse. On the other hand brainstem neurodegeneration has been suggested as an initial event in AD [9], and serotonergic dysfunction [11] as well as reductions in raphe neurones density have been reported in AD patients [29], depriving the hippocampal and cortical neurons from their critical modulatory influence [30]. Moreover specific reductions in serotonergic receptors densities are correlated to cognitive state in AD patients [31]. Nevertheless to our knowledge no previous studies have addressed whether specific serotonergic denervation could worsen AD pathology and associated cognitive impairment. Therefore we induced a selective serotonergic lesion in the RN of APPswe/PS1dE9 mice by injecting the selective serotonergic neurotoxin 5,7-DHT, previously used in other animal models to induce serotonergic denervation in rodents [21], [22], [23]. Serotonergic lesions were confirmed by tryptophan hydroxylase immunostaining in the RN and we observed a reduction in the number of labeled neurons, after 5,7-DHT treatment, both wildtype and APPswe/PS1dE9 mice, when compared with sham mice. Cortex and hippocampus also showed a depletion in the amount of tryptophan hydroxylase immunostaining supporting that projected regions where affected after selective serotonergic lesions. Although we cannot exclude that other neuronal populations could be affected by 5,7-DHT injections, previous administration of desipramin and nomifemsin to the mice should protect noradrenergic and dopaminergic neurons respectively. Following this idea, the fact that locomotor activity was not altered in lesioned mice also suggests that no other neuronal populations are affected.

Whereas it seems that drastic reduction of serotonergic innervation reduces anxiety behaviour in different paradigms [19], [32] it has also been reported that selective lesions of the medial prefrontal cortex serotonergic neurons increased anxiety-like behavior in rats [19], [32]. In our hands 5,7-DHT lesions of the RN in APPswe/PS1dE9 mice did not result in altered anxiety-like behaviour and these observations are in agreement with previous studies where 5,7-DHT was injected in rats dorsal raphe nucleus and no anxiety-related behaviour was observed [33]. To our knowledge the effect of 5,7-DHT lesions on anxiety-like behavior in mice has been little explored [34] and we can not exclude that higher or more widespread serotonergic denervation could lead to significant changes. However, the fact that no alterations were observed at this level, precludes the possibility that anxiety related behavior could interfere with learning and memory abilities. In this sense, cognitive assessment in the MWM test showed a significant impairment in 5,7-DHT lesioned transgenic mice, both during the acquisition and retention phases, supporting the role of the serotonergic system in learning and memory processes [8]. Following this idea, increasing serotonergic activity by chronic prophylactic administration of selective serotonin reuptake inhibitors (SSRI) improves cognition in 3×TgAD mice [35]. It has also been reported that serotonergic system role in cognition might be mediated by interacting with cholinergic, glutamatergic, dopaminergic or GABAergic neurotransmission [36]. Previous studies have shown some controversy regarding cognitive alterations after 5,7-DHT lesions [37], [38], nonetheless it seems that when serotonergic depletion is combined with other lesions, such as cholinergic denervation, cognitive deficits are worsened [39], [40], [41]. Our data are in accordance with these studies, and only when serotonergic lesion was combined with the presence of the APP/PS1 transgenes we could detect learning and memory dysfunction.

The close relationship between serotonergic and cholinergic systems has been widely assessed and both anatomical and functional interactions have been previously described (for review see [42], [43]). The complex modulation exerted by serotonin on the cholinergic system, both in the structures that project cholinergic innervation (nucleus basalis of Meynert, diagonal band of Broca and medial septum) and innervated areas (cortex and hippocampus) is highly dependent on the regions under study and the implicated serotonergic receptors. In this sense 5-HT2A, 5-HT4 and 5-HT7 receptors agonists seem to increase acetylcholine release, whereas 5-HT1A, 5-HT4 and 5-HT6 antagonists also increase acetylcholine release [42], [44], [45], [46]. Taking into account these considerations removing strong modulatory imputs, after 5,7-DHT lesion, to the hippocampus and cortex could potentially affect cholinergic innervation in these areas [30] and therefore we also analyzed cholinergic markers in the cortex and hippocampus. Although a slight reductions in ChAT immunostaining and AChE activity was observed we did not detect any significant effects. Whereas we can not exclude that further assessment of cholinergic system markers and activity could detect some alterations, as a consequence of the serotonergic RN denervation, to our knowledge the specific effect of 5,7-DHT lesions on the cholinergic system has not been explored.

Previous studies have shown that selective cholinergic denervation of the basal forebrain [6] or noradrenergic denervation of the locus coeruleus [47] can increase Aβ production and deposition as SP, and increased Aβ deposition has also been observed in stroke models induced in AD mice [48]. It has also been shown that Aβ plaques induced neurotoxicity may also stimulate sprouting of 5-HT fibers [49], and this might be considered an intrinsic protective mechanism in response to Aβ induced excitotocity in AD [42]. In this sense Cirrito et al. [50] have shown that AD patients on antidepressant treatment present significantly less amyloid load as quantified by positron emission tomography imaging with Pittsburgh Compound B. Similarly it has also been shown that prophylactic chronic administration of SSRI can reduce amyloid load in APPswe/PS1dE9 mice [50] and delay amyloid pathology in 3×TgAD mice [35]. Following this idea, it could be feasible that reducing serotonergic imputs after selective lesions, could increase Aβ deposition. To our knowledge this specific has not been explored, however in our study selective serotonergic lesions did not interfere with Aβ pathology in denervated areas, although our mice were lesioned when amyloid pathology is widely present in APPswe/PS1dE9 [14], as opposed to previously cited studies [35], [50]. In our hands, both TS stained dense-core plaques and anti-Aβ immunostaining, including also diffuse amyloid, were not affected by serotonergic denervation. Also soluble and insoluble Aβ40 and Aβ42 levels were similar in lesioned and sham APPswe/PS1dE9 groups, supporting the lack of effect of the selective serotonergic lesions on Aβ natural history. This might also relate to recent discussion about amyloid dependent and amyloid-independent stages of AD, and whereas in an initial phase disruption of the neuropil, loss of dendritic spines, remodeling of neurites, and inflammatory responses would derive from soluble oligomeric and fibrilar Aβ accumulation, the second phase would consist of the further development of tangles, synaptic and neuronal loss [51]. Although we cannot exclude an overall increase in SP deposition in other brain areas, or that longer postsurgical time or more extensive serotonergic lesions could lead to more dramatic changes, our data suggest that neurodegeneration of different neurotransmitter systems may play distinct roles in Aβ metabolism. We can not obviate that SP and Aβ levels only characterize parts of a complex combination of pathological features, and the presence of soluble microscopic oligomeric forms of Aβ, could be increased after the lesion, and could be responsible for detected cognitive alterations [52], since they likely contribute to the progressive neural system failure that occurs over decades [53].

On the other hand we detected a significant increase in tau phosphorylation in denervated areas, supporting previous studies that have pointed out to the possibility that serotonergic signaling may determine downstream protein phosphorylation [12]. Also, when prophylactic SSRI was administered to 3×TgAD mice a significant reduction of tau pathology has been observed [50]. In this sense it has also been shown that AD patients present a significant increase in the number of neurofibrillary tangles in the RN when compared with control patients [29], and although the authors did not detect a correlation between the presence of neurofibrillary tangles and behavioural changes, other studies in transgenic tau models of AD have reported consistent memory dysfunction in mice [54], [55]. Moreover it seems that earlier tau pathological stages, and not necessarily neurofibrillary tangles, are critical for the development of cognitive malfunctions [56]. Even though we detected a slight cognitive impairment, without increased SP deposition, our results are in accordance with previous studies where significant cognitive improvement has been detected by reducing tau pathology in AD models [57], [58].

To our knowledge the direct implication of the serotonergic system in Aβ or tau pathology has not been clarified, and the underlying explanation for the selective vulnerability of different brain regions in AD remains unclear, however our data point to the idea that different neurotransmitter systems may play specific roles in associated AD pathology. Since AD is a multisystem disorder affecting multiple neuronal populations including cholinergic, dopaminergic, glutamatergic or serotonergic systems, targeting individual systems would only improve the function of a subset of neuronal circuits affected in AD and therefore, no single therapy targeting an individual system would be able to effectively restore cognitive function or reduce behavioral abnormalities in people with AD [30]. Altogether, our data suggest that serotonergic denervation may increase tau hyperphosphorylation in denervated areas, and synergistically contribute to learning and memory impairment, as observed in AD, supporting the idea that therapeutic approaches could target specific neurotransmitter systems.
